# In the Absence of Type-1 IFN, HSV-1 LAT Increases γ34.5 Expression and Enhances Mortality in Infected Mice

**DOI:** 10.3390/v17081061

**Published:** 2025-07-29

**Authors:** Jay J. Oh, Ujjaldeep Jaggi, Deepak Arya, Shaohui Wang, Homayon Ghiasi

**Affiliations:** Center for Neurobiology and Vaccine Development, Ophthalmology Research, Department of Surgery, Cedars-Sinai Burns & Allen Research Institute, CSMC–SSB3, 8700 Beverly Blvd., Los Angeles, CA 90048, USA; jay.oh@cshs.org (J.J.O.); ujjaldeep.jaggi@cshs.org (U.J.); deepak.arya@cshs.org (D.A.); shaohui.wang@cshs.org (S.W.)

**Keywords:** type-1 IFN, survival, ICP35 (γ34.5), LAT, gB, ICP0, ICP4

## Abstract

Type-I Interferon (IFN) is essential for antiviral immunity in both mice and humans; thus, we investigated whether LAT affects HSV-1 infectivity in the absence of IFN by infecting IFNαβR^−/−^ and wild-type control mice with HSV-1 McKrae (LAT-plus) and dLAT2903 (LAT-minus) viruses. IFNαβR^−/−^ mice survived ocular infection with the LAT-plus virus, while no infected mice survived infection with the LAT-minus virus. Increased death in infected mice correlated with a higher expression in the neurovirulence γ34.5 gene but not with gB expression. To determine the region of LAT that contributed to higher mortality, IFNαβR^−/−^ mice were infected with recombinant viruses expressing the first 1.5 kb or the first 811bp region of 1.5 kb LAT. Similar to LAT-plus infected mice, IFNαβR^−/−^ mice infected with LAT1.5kb were protected from death, while infection with the LAT811bp virus was similar to that of LAT-minus, suggesting that increased pathogenicity in the absence of LAT depends on the second half of 1.5 kb LAT. To confirm the in vivo upregulation of γ34.5 expression in the absence of LAT, rabbit skin and Neuro2A cells were infected with LAT-plus, LAT-minus, LAT1.5kb, or LAT811bp viruses. γ34.5 expression was significantly higher in LAT-minus- and LAT811bp-infected rabbit skin cells and Neuro2A cells than in LAT-plus- and LAT1.5kb-infected cells, suggesting that sequences after the 811bp of LAT contribute to γ34.5 upregulation. However, except for γ34.5 expression, ICP0, ICP4, and gB expression were not affected by the absence of LAT or truncated forms of LAT. To confirm that higher γ34.5 expression contributes to higher mortality in the absence of LAT, we infected IFNαβR^−/−^ mice with a recombinant virus lacking LAT and γ34.5 expression, and, in contrast to LAT-minus, all infected mice survived. Our results suggest that LAT controls γ34.5 expression and that higher γ34.5 expression and mortality in infected mice are associated with the second half of 1.5 kb LAT.

## 1. Importance

The latency-associated transcript (LAT) of HSV-1 has multiple functions, including enhancing latency reactivation, anti-apoptotic activity, T cell exhaustion, and modulating host immune responses to infection. However, the role of LAT relative to type-1 interferon (IFN) signaling and its impact on virus pathogenicity are not well understood. Because IFN signaling affects LAT functions, we infected IFNαβR^−/−^ and wild-type mice with different doses of HSV-1 McKrae (LAT-plus), dLAT2903 (LAT-minus—lacking 2-kb LAT), dLAT-3.3A (has one copy of 1.5 kb LAT), dLAT2.6A (has the first 811 bp of 1.5 kb LAT), and dLAT2903-γ34.5 (lacks both 2-kb LAT and γ34.5) viruses. Our results showed for the first time that LAT is required to reduce mortality in the absence of IFN. The higher mortality in the absence of LAT is associated with increased γ34.5 gene expression as demonstrated in vivo. Thus, LAT controls neurovirulence by blocking upregulation of γ34.5 expression. Further, the region of LAT that is required to block γ34.5 expression is outside the region of LAT that contributes to higher latency reactivation and reduced apoptosis.

## 2. Introduction

Herpes simplex virus type-1 (HSV-1) infection is one of the most common ocular infections and a major cause of virus-induced blindness in the United States [1,2]. Following primary infection, the virus establishes lifelong latency in sensory neurons of infected hosts [3,4,5]. The latency-associated transcript (LAT) is the only abundant viral transcript detected in the neurons of infected hosts [3,4,6,7,8,9]. The primary 8.3 kb LAT transcript is spliced into a stable 2 kb LAT and an unstable 6.3 kb LAT [4,7,8,10]. LAT does not play a key role in initial infection or virulence, but is required to enhance latency and reactivation [11,12,13] in at least three ways: First, anti-apoptotic functions of LAT are essential to establish latency reactivation, and replacing LAT with other anti-apoptotic genes restores its latency and reactivation functions [14,15,16,17,18,19,20]. Second, LAT controls the expression of HSV-1 lytic cycle genes such as ICP0 and ICP4 to regulate productive viral infection [14,15,16,17,21]. And third, LAT represses expression of the cellular herpes virus entry mediator (HVEM) [22] and downregulates the type-1 interferon (IFN) pathway via the Janus kinase (JAK) pathway [23].

Type-I IFN is a major component of the innate immune response against viral infections and is essential for antiviral immunity in both mice and humans [24,25,26]. Some viruses can circumvent the IFN response to either prevent or block IFN signaling [27,28,29]. In both mice and humans, the IFN gene family has at least 20 comparable members [30,31,32,33,34]. There are at least 14 IFNα genes and a single IFNβ gene [30,31], and IFNα/β gene functions require their binding to the same receptor, a heterodimer composed of the transmembrane proteins IFNAR1 and IFNAR2 [35,36]. IFNα/β plays a significant role in HSV infection in vitro and in vivo, and HSV-1 can evade IFN responses through several mechanisms [37,38,39,40,41,42]. IFNαβR^−/−^ mice (also known as IFNAR1^−/−^ or CD118^−/−^) lack antiproliferative and antiviral responses associated with IFNα/β signaling [43,44]. Although the IFNα subtypes and IFNβ use the same IFNARs, they exhibit functional differences [45]. IFNαβR^−/−^ mice are highly susceptible to viral infection, including HSV-1 infection [39,43,44,46,47,48]. In contrast to highly susceptible IFNαβR^−/−^ mice, IFNα2A^−/−^ and IFNβ^−/−^ mice are less susceptible to HSV-1 infection [49,50]. Human studies have also shown that inborn genetic errors in IFN genes can damage the ability to control viral infections, leading to increased susceptibility to infectious disease [51,52,53,54].

Previously, we showed that LAT downregulates components of the IFN pathway [23]. LAT also interferes with ICP0 and ICP4 expression in infected cells [55,56]. Although, LAT promotes neuronal survival in the trigeminal ganglia (TG) of infected rabbits [14] and mice [20,57]. Neurons produce minimal IFNα/β in response to wild-type (WT) HSV-1 infection [58]. Several HSV-1 genes including ICP27 [59], ICP0 [60], US11 [61], virion host shutoff (vhs) [62], US3 [63], and ICP34.5 (i.e., γ34.5] [64] are known to inhibit IFNα/β signaling. Among these HSV-1 genes, the γ34.5 neurovirulence viral gene is central to countering several components of the host IFN response [65]. Two copies of γ34.5 exist in the HSV-1 genome repeat regions and are antisense to LAT [66]. Although it has been described as a late gene, γ34.5 counters the host response after late viral DNA synthesis as well as in the first hours of infection [67,68,69]. γ34.5 has been reported to specifically interfere with host shutoff of protein synthesis [70], inhibit autophagy by binding to Beclin 1 [71], and prevent activation of the IFN response through TANK-binding kinase (TBK1] [72].

**Figure 1:**
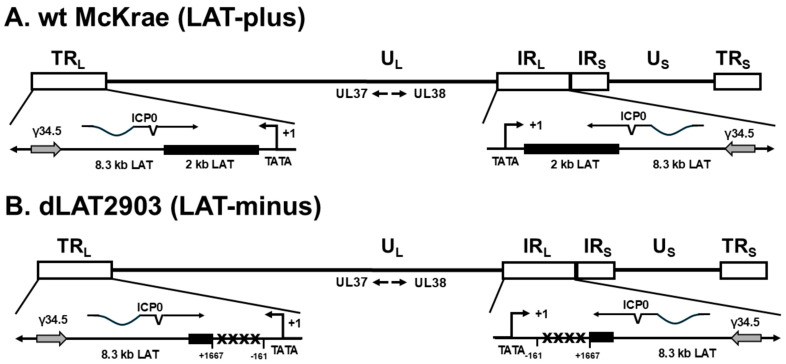

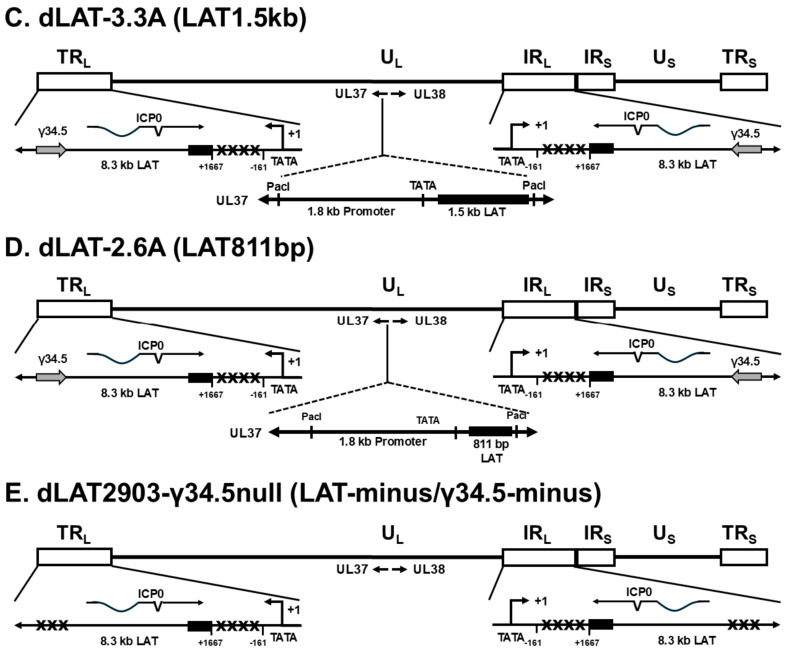
Construction of HSV−1 recombinant viruses. (**A**) WT HSV−1 strain McKrae genome in the prototypic orientation (LAT−plus). TR_L_ and IR_L_ represent the terminal and internal (or inverted) long repeats. TR_S_ and IR_S_ represent the terminal and internal (or inverted) short repeats. U_L_ and U_S_ represent the long and short unique regions, respectively. The solid rectangle represents the highly stable 2 kb LAT, while the gray arrows represent the ICP35, also known as the γ34.5 gene. The arrow at +1 indicates the start site for LAT transcription. (**B**) dLAT2903 (LAT−minus) has a deletion of LAT nucleotides −161 to +1667 relative to the start of LAT transcription in both copies of LAT. The XXXX indicates no LAT RNA synthesis [11]. (**C**) dLAT3.3A (LAT1.5kb) was derived from dLAT2903 by inserting the LAT promoter and DNA, encoding the first 1.5 kb of the 8.3 kb primary LAT transcript into the unique long region between the UL37 and UL38 genes of HSV−1 [12]. (**D**) Similar to dLAT3.3A, dLAT2.6A (dLAT−811bp) retains the dLAT2903 deletion in both copies of LAT and differs from dLAT2903 only in that it contains 811 bp of the 1.5 kb LAT inserted into the viral unique long region between UL37 and UL38 rather than the normal LAT location. Like dLAT2903 (LAT−minus) and dLAT3.3A (LAT1.5kb), dLAT2.6A (LAT811bp) cannot make LAT transcripts from the normal LAT location in the viral long repeats. Instead, dLAT2.6A transcribes only the first 811 nt of the primary 8.3 kb LAT [73]. (**E**) dLAT2903−γ34.5 null virus does not transcribe LAT or the γ34.5 gene [74]. Throughout the study, McKrae, dLAT2903, dLAT3.3A, and dLAT2.6A viruses are referred to as LAT−plus, LAT−minus, LAT1.5kb, and LAT811bp, respectively.

**Figure 2:**
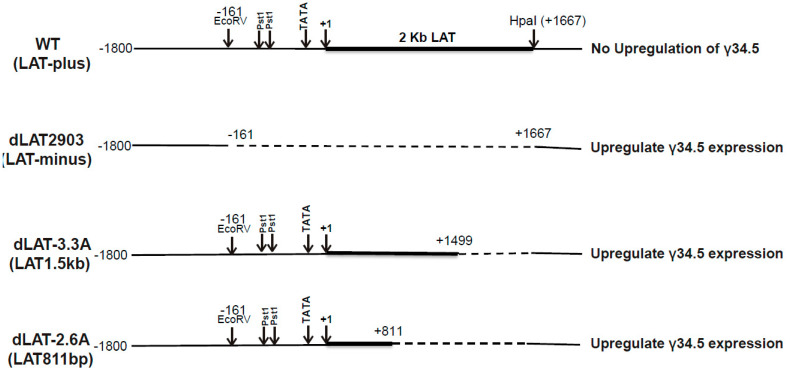
Detailed schematic diagram of LAT regions used to make recombinant viruses. WT includes full−length 2 kb LAT between −161 nt and +1667 nt. The arrow at +1 indicates the start site for LAT transcription. In dLAT2903, the LAT region between −161 nt and +1667 nt was deleted. In dLAT3.3A (LAT1.5kb), the LAT region between −161 nt and +1499 nt was deleted. In dLAT2.6A (LAT811bp), the LAT region between −161 nt and +811 nt was deleted. The effect of LAT on γ34.5 expressions are shown on the right side of figure.

LAT regulates immune responses to infection, and no other studies have evaluated the inter-relationship of LAT with IFNs and γ34.5 in vivo or in vitro. To study these relationships, we used IFNαβR^−/−^ mice that lack functional IFN expression along with WT HSV-1 strain McKrae (LAT-plus), and four HSV-1 recombinant viruses derived from WT McKrae (LAT-plus), and their schematic diagrams are shown in Figure 1 and Figure 2 as follows: dLAT2903 (LAT-minus, lacking the 2-kb LAT), dLAT3.3A (expressing one copy of the first 1.5 kb of LAT inserted between the UL37 and UL38 genes), dLAT2.6A (expressing one copy of the first 811 bp of LAT inserted between UL37 and UL38 genes), and dLAT2903-γ34.5 null (similar to dLAT2903, lacking both 2 kb of LAT as well as the γ34.5 gene). Our results suggest that: (1) all IFNαβR^−/−^ mice survived infection with 1 × 10^2^ pfu/eye of LAT-plus virus, while none of the dLAT2903-infected mice survived infection at this dose; (2) survival was similar in LAT1.5kb and LAT-plus infected mice, while mice infected with LAT811bp and LAT-minus also showed similar survival, suggesting that increased survival in LAT-plus and LAT1.5kb viruses requires the first 811 bp of the 1.5 kb LAT; (3) deleting the γ34.5 gene in the absence of LAT increased survival to that of the LAT-plus virus; (4) in both rabbit skin (RS) and Neuro2A-infected cells, γ34.5 expression was significantly higher in LAT-minus- and dLAT2.6A-infected cells than in LAT-plus- and LAT1.5kb-infected cells; (5) similar to in vitro results, γ34.5, but not gB expression, was higher in TG, brain, and brainstem of IFNαβR^−/−^ mice infected with the LAT-minus virus than in those infected with the LAT-plus virus; and (6) except for γ34.5 expression, ICP0, ICP4, and gB expression were not affected by the presence or absence of LAT in infected RS or Neuro2A cells. Overall, the results of this study showed that higher γ34.5 expression in the absence of LAT correlated with higher mortality in infected mice. Despite the presence of anti-apoptotic LAT functions in the first 811 bp of 1.5 kb LAT, functions in the second half of the 1.5 kb LAT correlate with higher mortality and higher γ34.5 expression in infected mice.

## 3. Results

### 3.1. LAT Protects Against Neurovirulence in the Absence of Type-1 Interferons

In numerous studies over the past 40 years, we have infected C57BL/6 mice from the Jackson Laboratory with McKrae (LAT-plus) and dLAT2903 (LAT-minus) viruses and observed more deaths in mice infected with the LAT-minus virus than in those infected with the LAT-plus virus. However, this trend never reached significant differences even after combining many experiments, which may be because LAT can suppress components of the IFN pathway in infected mice [23]. To identify contributions of IFNs to our WT mice observations in the absence of LAT, we infected IFNαβR^−/−^ mice with 1 × 10^4^, 1 × 10^3^, and 1 × 10^2^ pfu/eye of McKrae (LAT-plus) and dLAT2903 (LAT-minus, lacking the 2-kb LAT) viruses (Table 1) (Figure 1 and Figure 2). The WT C57BL/6 control group was infected with one dose (1 × 10^4^ pfu/eye) of each virus (Table 1, WT). Survival of ocularly infected mice was recorded for 28 days. All WT mice infected with 1 × 10^4^ pfu/eye of either LAT-plus or LAT-minus viruses survived ocular infection (100% survival), while IFNαβR^−/−^ mice infected with the same dose of LAT-plus or LAT-minus did not survive (0% survival) (Table 1). At a dose of 1 × 10^3^ pfu/eye, 9 of 16 of IFNαβR^−/−^ mice infected with LAT-plus survived ocular infection (56% survival), while only 1 of 16 IFNαβR^−/−^ mice infected with LAT-minus virus survived ocular infection (6% survival). These differences were statistically significant (Table 1, 1 × 10^3^, *p* < 0.001, Chi-square). At a dose of 1 × 10^2^ pfu/eye, 10 of 10 IFNαβR^−/−^ mice infected with the LAT-plus virus survived infection (100% survival), while 0 of 10 IFNαβR^−/−^ mice infected with the LAT-minus virus survived infection (0% survival) (Table 1, 1 × 10^2^, *p* < 0.0001, Chi-square). Thus, despite the higher sensitivity of IFNαβR^−/−^ mice to HSV-1 infection, LAT appears to protect against mortality in infected mice without inducing type I IFN expression.

### 3.2. The γ34.5 Gene Is Upregulated in the Absence of LAT in LAT-Minus-Infected RS and Neuro2A Cells in Vitro

**Figure 3:**
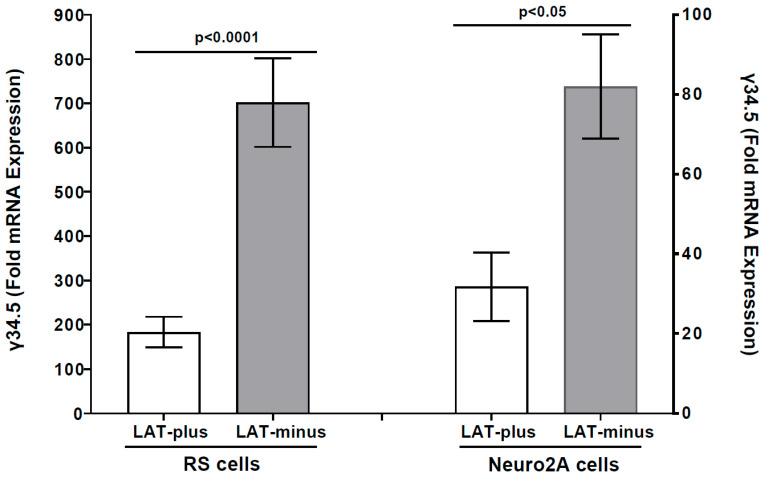
Effect of LAT on γ34.5 expression in vitro. RS and Neuro2A cells were infected with LAT−plus or LAT−minus viruses at one pfu/cell for 24 h. TRIzol was added to infected-cell lysates, and total RNA was extracted and amplified by qRT−PCR using γ34.5 primers. The 2^−ΔΔ*CT*^ method was used to calculate fold changes in γ34.5 gene expression relative to expression in uninfected controls. Experiments were repeated twice. Each bar represents the mean ± SEM of 6 samples from two experiments.

LAT-minus-infected IFNαβR^−/−^ mice that do not express LAT have higher mortality than LAT-plus infected mice (see Table 1). Because γ34.5 has been shown to contribute to HSV-1 neurovirulence [65], we investigated whether the increased mortality observed in LAT-minus mice, in the absence of LAT, is associated with γ34.5 expression. RS cells were infected with one pfu/cell of LAT-plus or LAT-minus virus for 24 h, and γ34.5 expression was measured using qRT-PCR. We found significantly higher γ34.5 expressions in LAT-minus-infected RS cells than in LAT-plus-infected cells (Figure 3, *p* = 0.0011, RS cells). We next asked whether the absence of LAT affects γ34.5 expression in Neuro2A cells infected with viruses that express either LAT-plus or LAT-minus. Similar to RS cells, γ34.5 expression was significantly higher in LAT-minus-infected Neuro2A cells than in LAT-plus-infected Neuro2A cells (Figure 3, *p* < 0.05, Neuro2A cells). Despite higher γ34.5 expression in both LAT-minus-infected RS and Neuro2A cells than in uninfected cells, γ34.5 expression was lower in infected Neuro2A cells than in infected RS cells for both LAT-plus and LAT-minus viruses. Both of these viruses infect RS cells more efficiently than Neuro2A cells, suggesting that γ34.5 is upregulated in the absence of LAT.

**Figure 4:**
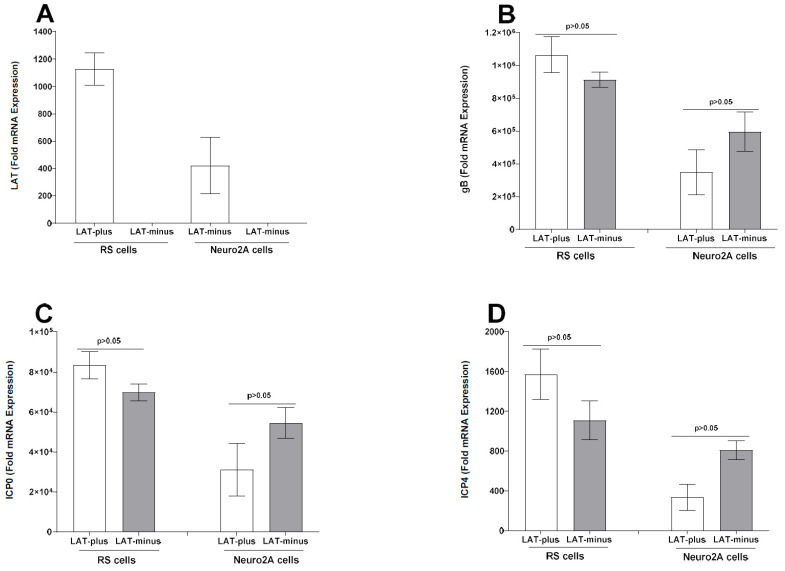
Absence of LAT sequences do not affect expression of gB, ICP0, and ICP4 transcripts in vitro. Total RNA from infected RS and Neuro2A cells (see Figure 3) was used to measure the expression of LAT, gB, ICP0, and ICP4 mRNA by quantitative real−time PCR (qRT−PCR) using specific primer sets for each gene. Each bar represents the mean ± SEM of 6 samples from two independent experiments.

To determine if, similar to γ34.5, absence of the LAT sequence affects expression of other viral genes, levels of LAT, gB, ICP0, and ICP4 expression were measured in infected RS cells as described above (Figure 3). As expected, LAT expression was only seen in LAT-plus-infected cells, not in LAT-minus-infected RS cells (Figure 4A, RS cells). In contrast to γ34.5 expression, no differences in gB (Figure 4B, *p* > 0.05, RS cells), ICP0 (Figure 4C, *p* > 0.05, RS cells), or ICP4 (Figure 4D, *p* > 0.05, RS cells) gene expressions were detected in LAT-plus- and LAT-minus-infected groups. Thus, while the absence of LAT upregulated γ34.5 gene expression, it did not affect gB, ICP0, or ICP4 expression in RS cells. Similar to RS cells, LAT expression was only detected in LAT-plus-infected Neuro2A cells and not in LAT-minus-infected cells (Figure 4A, Neuro2A cells). Further, no differences in gB (Figure 4B, *p* = 0.2), ICP0 (Figure 4C, *p* = 1), or ICP4 (Figure 4D, *p* = 0.2) gene expressions were seen in Neuro2A cells infected with LAT-plus or LAT-minus viruses. These results show that the absence of LAT did not affect the expression of gB, ICP0, or ICP4 in infected RS and Neuro2A cells.

Overall, our results with LAT-plus- and LAT-minus-infected cells suggest that the absence of LAT affects γ34.5 expression in both RS- and Neuro2A-infected cells, but not gB, ICP0, or ICP4 expression. Thus, higher γ34.5 expression in vitro correlates with higher mortality in the absence of LAT.

### 3.3. Upregulation of γ34.5, but Not gB, in the Absence of LAT in the CNS of Infected Mice

**Figure 5:**
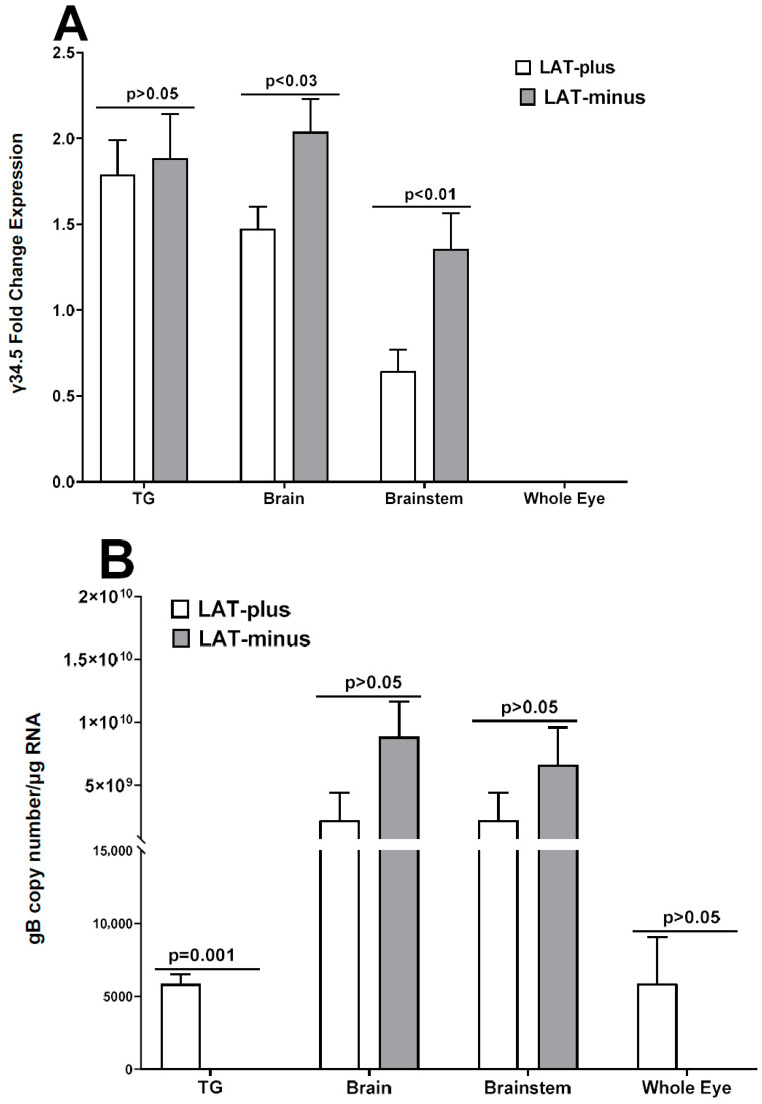
γ34.5 and gB transcripts in TG, brains, brainstems, whole eyes, and after ocular infection. IFNαβR^−/−^ mice were infected ocularly with 1 × 10^2^ pfu/eye of LAT−minus or LAT−plus viruses as described in Materials and Methods. After mice were euthanized on the indicated days, TG, brains, brainstems, and whole eyes were removed on days 3 and 5 PI, and homogenized and total RNA were extracted. γ34.5 and gB expression were measured by qRT−PCR and normalized with GAPDH. Relative gB RNA copy numbers were calculated using standard curves generated from plasmid pAc-gB1 [75]. The 2^−ΔΔ*CT*^ method was used to calculate fold changes in γ34.5 gene expression relative to expression in uninfected controls. Each point represents the mean ± SEM of 6 samples from two independent experiments.

In vitro results above (Figure 3 and Figure 4) suggest that the absence of LAT increases γ34.5 expression in LAT-minus-infected RS and Neuro2A cells. Because the γ34.5 neurovirulence gene contributes to higher mortality [65,67,68], we next investigated whether the higher mortality in IFNαβR^−/−^ mice infected with the LAT-minus virus compared to those infected with LAT-plus virus correlates with increased γ34.5 gene expression in the CNS of infected mice. IFNαβR^−/−^ mice were infected with 1 × 10^2^ pfu/eye of LAT-minus or LAT-plus viruses as described above and in Materials and Methods. On days 3 and 5 post infection (PI), infected mice were sacrificed to collect TG, brains, brainstems, and eyes. Total RNA was analyzed for γ34.5 expression with gB expression as a control (Figure 5). On day 3 PI, gB, and γ34.5 expressions were not detected in the eye, TG, brainstem, or brain of infected mice. gB and γ34.5 expression were both seen in TG, brainstem, and brain of infected mice (Figure 5). TG of LAT-plus- and LAT-minus-infected mice expressed similar levels of γ34.5 (Figure 5A, *p* = 0.8, TG), but significantly higher γ34.5 expression was seen in the brainstem (Figure 5A, *p* = 0.03, brainstem) and brain (Figure 5A, *p* = 0.01, brain) of LAT-minus-infected mice than in LAT-plus-infected mice. In contrast, no γ34.5 expression was detected in the whole eyes from LAT-minus- and LAT-plus-infected mice (Figure 5A, whole eyes). While gB expression in TG of LAT-plus-infected mice was significantly higher than in LAT-minus-infected mice (Figure 5B, *p* = 0.001, TG), gB expression in brainstem (Figure 5B, *p* = 0.09, brainstem) and brain (Figure 5B, *p* = 0.2, brain) of LAT-plus- and LAT-minus-infected mice was similar. Although γ34.5 expression was not detected in whole eyes from infected mice (Figure 5A), gB was detected in whole eyes of both LAT-plus- and LAT-minus-infected mice. Differences were not statistically significant (Figure 5B, *p* = 0.1, whole eyes). These results suggest that the higher mortality of LAT-minus-infected mice than LAT-plus-infected mice was due to higher γ34.5 expression rather than higher virus replication in the CNS of infected mice.

### 3.4. Mapping the Neurovirulence Region of LAT That Contributes to Increased Mortality and γ34.5 Upregulation in Infected IFNαβR^−/−^ Mice

As described above, our results with LAT-plus and LAT-minus viruses suggested that the absence of LAT contributes to higher mortality in infected IFNαβR^−/−^ mice. To determine which region of LAT contributes to increased neurovirulence in LAT-minus infected mice, IFNαβR^−/−^ mice were infected ocularly with 1 × 10^4^, 1 × 10^3^, and 1 × 10^2^ pfu/eye of dLAT3.3A (expressing 1.5 kb of LAT) and dLAT2.6A (expressing the first 811 bp of 1.5 kb LAT) viruses, and WT C57BL/6 control mice were infected only with 1 × 10^4^ pfu/eye of each virus (Table 2) (Figure 1 and Figure 2). Survival of ocularly infected mice was recorded for 28 days. All WT mice infected with 1 × 10^4^ pfu/eye of LAT1.5kb or LAT811bp viruses survived ocular infection, while 100% of IFNαβR^−/−^ mice infected with the same dose of these viruses died (Table 2, 1 × 10^4^ pfu/eye). At a dose of 1 × 10^3^ pfu/eye, 3 of 9 (33%) mice infected with LAT1.5kb survived ocular infection, while 0 of 8 infected with LAT811bp survived ocular infection. These differences were statistically significant (Table 2, 1 × 10^3^, *p* < 0.05, Chi-square). At a dose of 1 × 10^2^ pfu/eye, 9 of 9 (100%) mice infected with LAT1.5kb survived infection, while 10 of 10 (100%) mice infected with the LAT811bp virus died (Table 2, 1 × 10^2^ pfu/eye, *p* < 0.001, Chi-square). Thus, in contrast to the LAT-plus virus, which expresses two copies of LAT, the ability of LAT1.5kb and LAT811bp viruses, which express only one copy of LAT, to protect against mortality is located after the 811 bp of the 1.5 kb LAT.

### 3.5. γ34.5 Gene Is Upregulated in the Absence of the Second Half of LAT in LAT811bp-Infected RS and Neuro2A Cells In Vitro

**Figure 6:**
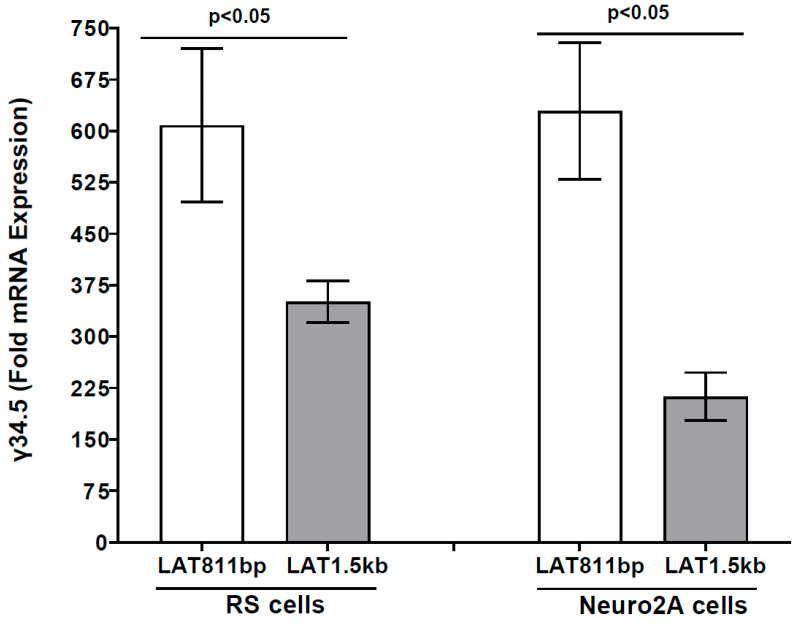
Effects of truncated LAT on γ34.5 expression. RS and Neuro2A cells were infected with 1 pfu/cell of LAT811bp or LAT1.5kb viruses. Infected cells were collected at 24 h PI, total RNA was extracted, and qRT−PCR was performed to quantify γ34.5 RNA levels. Results are shown as mean ± SEM of 6 samples from two separate experiments.

Survival studies reported in Table 2 for LAT1.5kb and LAT811bp showed that LAT1.5kb behaves like the WT McKrae virus (LAT-plus), while LAT811bp behaves like the LAT-minus virus. To determine if truncated LAT sequences affect γ34.5 expression, RS cells were infected with LAT811bp or LAT1.5kb viruses as described above, and γ34.5 expression was determined in LAT1.5kb- and LAT811bp-infected RS cells. Similar to the LAT-minus virus described above (Figure 3), γ34.5 expression was higher in LAT811bp-infected RS cells than in LAT1.5kb-infected cells (Figure 6, *p* < 0.05). To evaluate γ34.5 expression in Neuro2A cells, they were infected with LAT1.5kb or LAT811bp viruses for 24 h, and γ34.5 transcripts were measured by qRT-PCR. γ34.5 expression was significantly higher in LAT811bp-infected Neuro2A cells than in LAT1.5 kb-infected cells (Figure 6, *p* = 0.003), suggesting that LAT811bp behaves like LAT-minus, with the LAT region involved in upregulating γ34.5 and higher mortality located after bp 811 of the 1.5 kb LAT. However, in contrast to higher γ34.5 expression in RS cells infected with LAT-plus and LAT-minus than in Neuro2A cells (Figure 3), γ34.5 expression was similar in RS and Neuro2A cells infected with LAT1.5kb and LAT811bp.

**Figure 7:**
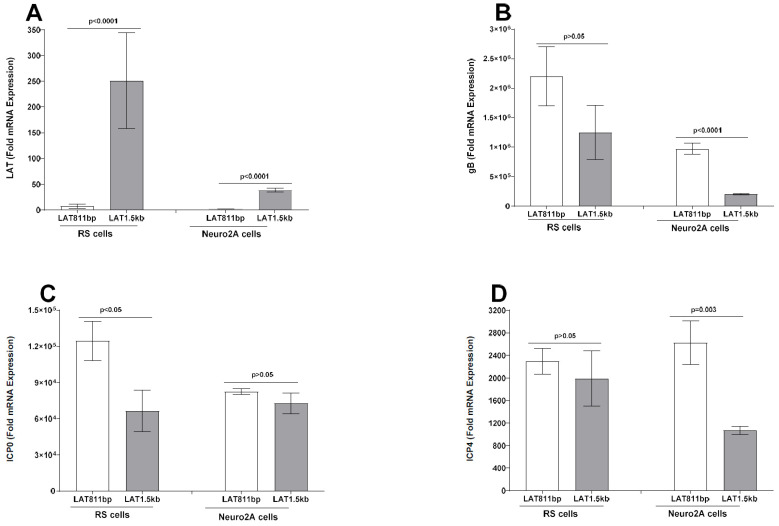
Effects of truncated LAT on expression of gB, ICP0, and ICP4 transcripts. Total RNA from RS and Neuro2A cells infected with LAT811bp or LAT1.5kb viruses (see Figure 5) was used to measure gene expression of LAT, gB, ICP0, and ICP4 by quantitative real-time PCR (qRT−PCR). Each bar represents the mean ± SEM of 6 samples from two separate experiments.

Expression levels of LAT, gB, ICP0, and ICP4 transcripts from RS and Neuro2A cells infected with LAT1.5kb and LAT811bp viruses were also determined by qRT-PCR. As expected, LAT expression was higher in RS cells infected with the LAT1.5kb virus than with the LAT811bp virus (Figure 7A, *p* < 0.0001, RS cells). Although expressions of gB (Figure 7B, *p* = 0.2, RS cells) and ICP4 (Figure 7D, *p* = 0.6, RS cells) did not differ, ICP0 expression was significantly higher in RS cells infected with LAT811bp than in LAT1.5kb-infected RS cells (Figure 7C, *p* = 0.03, RS cells). Similar to RS cells, LAT expression was also higher in Neuro2A cells infected with the LAT1.5kb virus than with the LAT811bp virus (Figure 7A, *p* < 0.0001, Neuro2A cells). Expressions of gB (Figure 7B, *p* < 0.0001, Neuro2A cells) and ICP4 (Figure 7D, *p* < 0.003, Neuro2A cells) were significantly higher in LAT811bp-infected Neuro2A cells than in LAT1.5kb-infected cells. In contrast, ICP0 expression did not differ significantly in Neuro2A cells infected with LAT1.5kb or LAT811bp viruses (Figure 7C, *p* = 0.3, Neuro2A cells).

### 3.6. Upregulation of γ34.5 in the Absence of LAT Affects Survival in Infected Mice

The above results using both in vivo and in vitro experiments suggest that LAT suppression of γ34.5 expression reduces death in IFNαβR^−/−^ mice. As a proof of concept, IFNαβR^−/−^ mice were infected with 1 × 10^4^, 1 × 10^3^, and 1 × 10^2^ pfu/eye of a recombinant virus lacking both LAT and γ34.5 genes (dLAT2903-γ34.5null). Similar to the results with LAT-plus viruses shown in Table 1 and Table 2, at 1 × 10^4^ pfu/eye all infected mice died, at 1 × 10^3^ pfu/eye 40% (2 of 5) of infected mice survived, while at 1 × 10^2^ pfu/eye all infected mice survived (Table 2, dLAT2903-γ34.5null virus). These results confirm our overall hypothesis that LAT contributes to higher expression of the γ34.5 neurovirulence gene, thus increasing mortality in infected IFNαβR^−/−^ mice.

## 4. Discussion

Previous studies have reported that HSV-1 LAT is expressed in both acute and latent infections and has multiple functions, including (1) suppressing the expression of lytic genes, such as the immediate early genes ICP0 and ICP4 [56,76]; (2) enhancing latency reactivation in an infected host [11]; (3) anti-apoptotic functions that contribute to enhancing latency reactivation [14,15,16,17,18,19,20]; (4) downregulating the type-1 IFN pathway during latency [23,77]; (5) encoding two sncRNAs that interact with HVEM and affect latency reactivation in infected hosts [78,79,80]; and (6) encoding multiple microRNAs, but their contributions to HSV-1 pathogenesis are unclear [81,82,83,84]. However, the role of LAT in neurovirulence in the absence of antiviral immunity remains unclear. Thus, in this study, we evaluated the effects of LAT on HSV-1 infectivity using IFNαβR^−/−^ mice, which lack type-1 IFN responses, along with HSV-1 recombinant viruses that either lack LAT or express different regions of LAT in vivo and in vitro.

The increased mortality in IFNαβR^−/−^ mice infected with the LAT-minus (dLAT2903) virus, even at 1 × 10^2^ pfu/eye, suggested the importance of LAT in protecting against increased mortality in the absence of type I IFN. Increased mortality in LAT-minus-infected IFNαβR^−/−^ mice correlated with higher γ34.5 expression in the TG, brain, and brainstem than that in LAT-plus-infected mice. However, similar gB expression in LAT-plus- and LAT-minus-infected mice suggested that higher mortality is associated with increased expression of the γ34.5 neurovirulence gene rather than higher virus replication in LAT-minus-infected mice. These results indicate that LAT may prevent enhanced γ34.5 and host innate immune responses in the absence of type-1 IFN expression. Thus, the higher expression of the γ34.5 gene, a major neurovirulence gene, could contribute to higher mortality in the absence of LAT expression. Conversely, the absence of γ34.5 may make the virus less neurovirulent in the infected host [65,67,68]. Herpes simplex encephalitis (HSE) is the most common form of viral encephalitis caused by HSV-1, and in this study, despite similar levels of gB expression in the CNS of infected mice, all infected mice in the LAT-minus and LAT811bp groups died between days 6 and 7 with a hunched posture and minimal movement, typical symptoms of HSE. Thus, since type-1 IFN has both antiviral and anti-inflammatory functions, the higher mortality in the absence of LAT could be due to increased inflammatory responses in infected mice, as well as higher γ34.5 expression. Similar to our in vivo results in RS and Neuro2A cells, γ34.5 expression was higher in the absence of 2 kb LAT and in the absence of the second half of 1.5 kb LAT. Our in vitro and in vivo results suggest that higher γ34.5 expression correlates with the absence of 2 kb LAT or truncated LAT. However, ICP0, ICP4, and gB expressions were not affected by the presence or absence of LAT, suggesting that higher γ34.5 expression is independent of other viral transcripts both in vivo and in vitro. To confirm our in vitro results that higher γ34.5 expression was associated with more mortality in infected mice, we looked at survival in mice lacking both LAT and γ34.5 expression. As expected, and in contrast to the LAT-minus–γ34.5-plus virus, all mice infected with LAT-minus and γ34.5-minus viruses survived infection with 1 × 10^2^ pfu/eye of the infected virus, suggesting that LAT affects γ34.5 expression and thus neurovirulence. HSV-2 has been reported to target γ34.5 expression by producing the viral microRNA, which is expressed from the LAT exon 2 [85]. These authors concluded that the microRNA was abundantly detected in the ganglia of latently infected guinea pigs and was shown to reduce γ34.5 expression specifically. Additionally, microRNAs generated from the HSV-2 LAT region and microRNAs produced from LAT that target γ34.5 expression were conserved in HSV-1 [85]. Thus, similar to the HSV-2 study, our results from the current HSV-1 study showed that the absence of 2 kb LAT in the LAT-minus virus increased the suppressive effects of LAT on γ34.5 expression in vitro and, by extension, increased mortality in vivo, possibly due to increased inflammation and/or cytokine storm.

Both LAT and γ34.5 are located in long repeats of the HSV-1 genome and therefore are present in two copies per genome [66]. The primary LAT transcript is 8.3 kb, and gives rise to a family of LAT RNAs (LATs), including a very stable 2 kb LAT that appears to be an intron spliced from the primary transcript [7,8,86]. The LAT-minus (dLAT2903) virus, which lacks the 2 kb stable LAT, has less latency and reactivation in rabbits and mice [11,73], and has more apoptosis [14] than the WT McKrae (LAT-plus) virus. We have shown that expressing the first 1.5 kb of the 2 kb LAT is sufficient for WT levels of spontaneous reactivation using the LAT1.5kb recombinant virus, even when the virus expresses only one copy of an LAT fragment [12]. This recombinant virus completely restored WT levels of spontaneous reactivation to the LAT1.5kb mutant virus. Later, it was shown that the spontaneous reactivation function of LAT is within the first 811 bp of the 1.5 kb LAT (i.e., dLAT2.6A virus) [73]. In our current study to fine map the region of LAT that contributes to increased γ34.5 expression in both RS and Neuro2A cells, we infected these two cell types with LAT1.5kb and LAT811bp viruses. Our results showed that the ability of LAT1.5kb to reduce γ34.5 expression was similar to that of WT McKrae (LAT-plus). In contrast, the ability of LAT811bp to upregulate γ34.5 expression was identical to the dLAT2903 (LAT-minus) virus. These results suggest that the LAT region involved in increasing γ34.5 expression in vitro and increasing mortality in infected mice is located in the second half of the 1.5 kb LAT. Previously, it was shown that anti-apoptotic LAT functions are located in the first 811 bp of LAT [12,14,73]. Thus, higher mortality in LAT-minus viruses is independent of LAT anti-apoptotic function. LAT has been shown to play a role in heterochromatin assembly, and a mutant virus lacking the 1.8 kb LAT region showed reduced levels of H3K27me3 and H3K9me3 during latency [87]. However, the effect of various LAT mutant viruses in the absence of type-1 IFN during primary infection may not be associated with histone modifications. HSV-1 encodes at least 16 microRNAs (miRNAs) [81], while there are at least 6 miRNAs within the 8.3 kb LAT, but no miRNA is detected within the 2 kb LAT [88]. Thus, miRNAs do not play any role in higher mortality in the absence of stable LAT in infected mice.

In the absence of LAT, increased mortality in mice infected with the LAT-minus virus was associated with higher γ34.5 expression in the CNS of infected mice. This increased mortality occurred despite similar levels of gB transcript in the CNS of LAT-minus and LAT-plus-infected mice, suggesting that increased mortality is associated with the increased expression of the γ34.5 neurovirulence gene. In this study, we also demonstrated that, similar to LAT-plus-infected mice, in the absence of both the LAT and γ34.5 genes, all infected mice survived ocular infection. In our study, the presence of LAT affected γ34.5 gene expression in the absence of type-1 IFN in mice infected with dLAT2903 (LAT-minus) but not in McKrae (LAT-plus)-infected mice. These results indicate that LAT plays an additional role during HSV-1 infection. In terms of the host responses [22,23,89,90,91,92,93,94,95], published studies have generated compelling evidence that, in contrast to T cells, macrophages, IFNγ, and DCs, LAT modulates the effects of type-I IFN response in infected mice. These results are in line with the current studies that show type-1 IFN knockout mice are more sensitive to infection with LAT-minus viruses than LAT-plus WT McKrae virus.

In conclusion, the results of this study establish a new role for LAT in regulating type-1 IFN and, consequently, the γ34.5 neurovirulence gene. Collectively, the absence of LAT enhanced γ34.5 expression in vitro, indicating that LAT plays a protective role in vivo. As a result, the absence of LAT led to higher γ34.5 expression, which consequently increased mortality in infected mice. Using a series of recombinant viruses that lack LAT or contain truncated parts of LAT in this study, we report that the second half of the 1.5 kb LAT increased both mortality and γ34.5 expression in infected mice. The importance of γ34.5 upregulation in the absence of LAT was further confirmed using a recombinant virus lacking both LAT and γ34.5 expression. To recap, this report’s results suggest a synergistic role of LAT and γ34.5 in HSV-1 infection, as well as enhanced neurovirulence in the absence of type-1 IFN encoded within the second half of the 1.5 kb LAT. Despite the seriousness of the recurrence of HSV-1 and the potential cause of the development of life-threatening HSE, there are currently no FDA-approved medical countermeasures or therapeutic strategies available to suppress the neuropathogenesis of HSV-1. Thus, the new findings of this study highlight the critical need for the development of alternative approaches to prevent the recurrence of HSE associated with HSV-1. Therefore, the discovery of a new crucial feature of LAT participating in the regulation of neuropathogenesis of HSV-1 is a necessary first step in developing strategies for prevention or therapeutics for the devastating HSV-1-associated neurological disease.

## 5. Materials and Methods

### 5.1. Ethics Statement

All animal procedures were performed in strict accordance with the Association for Research in Vision and Ophthalmology Statement for the Use of Animals in Ophthalmic and Vision Research and the NIH Guide for the Care and Use of Laboratory Animals (ISBN 0-309-05377-3). Animal research protocols were approved by the Institutional Animal Care and Use Committee of Cedars-Sinai Medical Center (Protocols #8837).

### 5.2. Structure of Recombinant Viruses

HSV-1 McKrae (LAT-plus) was used as the parental virus to construct all recombinant viruses used in this study [11,12,17,73,74]. Schematic diagrams of WT McKrae and detailed construction of the four McKrae-derived recombinant viruses are shown in Figure 1 and Figure 2 and described in the Figure 1 and Figure 2 legends.

### 5.3. Cell Lines and Mice

Plaque-purified HSV-1 strains of the McKrae and McKrae-derived recombinant viruses were grown in rabbit skin (RS) cell monolayers in minimal essential medium (MEM) containing 5% fetal bovine serum (FBS), as described previously [11,96]. Neuro2A cells (CCL 131, American Type Culture Collection) were maintained in Dulbecco’s modified Eagle medium (DMEM) with 10% FBS. Neuro2A has competent interferon responses and, as they are susceptible to HSV-1 infection, they play a crucial role in regulating the expression of IFN-α/β genes, thereby generating an antiviral response. Neuro2A cells were used for these studies because we previously found that LAT had a positive effect on cell survival in these cell lines and are used as a representative tissue culture of neuronal type cell for studying LAT’s effect on apoptosis. These cell lines serve as a model to investigate how LAT’s anti-apoptotic function, a key aspect of immune evasion by viruses such as Herpes Simplex Virus, is manifested at the cellular level. Neuro2a cells synthesize large amounts of microtubules, which are involved in various neuronal processes, such as axonal transport and neuronal development. This makes them useful for studying how proteins like LAT might influence these processes. RS is a primary rabbit skin cell line, and HSV-1 grows on them very efficiently. Type-1-interferon-receptor-deficient (IFNαβR^−/−^) mice (6–8 weeks of age, both sexes) in the C57BL/6 background and WT C57BL/6 mice were purchased from the Jackson Laboratory (Bar Harbor, ME) and bred in-house.

### 5.4. Ocular Infection and Survival of Infected Mice

Mice were randomly infected ocularly with 1 × 10^4^, 1 × 10^3^, and 1 × 10^2^ pfu/eye of each virus. A total of 129 IFNαβR-/- and 41 WT C57BL/6 mice were used in the study, and the number of mice per dose of infection ranged from 5 to 16 mice. Each virus was suspended in 2 μL of tissue culture media and administered as an eye drop without prior corneal scarification. All IFNαβR^−/−^ mice infected with LAT-minus viruses died by day 10 post infection. We also performed eye swabs in mice infected with 100 pfu/eye of each virus, but no infectious virus was detected by standard plaque assay in the eyes of infected mice. However, viral transcripts in the eye, TG, brain, and brainstem of infected mice are described below.

### 5.5. In Vitro Infection of RS and Neuro2A Cells

RS and Neuro2A cell monolayers were infected with 1 pfu/cell of LAT-plus, LAT-minus, LAT1.5kb, or LAT811bp for 24 h. TRIzol (Qiagen, Hilden, Germany) was added to infected cell lysates, and cells were harvested and stored at −80 °C until processing.

### 5.6. RNA Extraction, cDNA Synthesis, and TaqMan RT-PCR

Isolation and purification of total RNA from infected RS and Neuro2A cell lysates described above were performed using RNeasy columns (Qiagen, Hilden, Germany) as we described previously [97,98]. Following RNA extraction, 700 ng of total RNA was reverse-transcribed using random hexamer primers and murine leukemia virus reverse transcriptase using a high-capacity cDNA reverse transcription kit (Applied Biosystems, Foster City, CA, USA). Quantitative real-time PCR (qRT-PCR) was performed using the TaqMan gene expression assay kit in 384-well plates on an ABI QuantStudio 5 (Applied Biosystems, Forster City, CA, USA). Expression of LAT, gB, ICP0, and ICP4 were determined using the following custom-made primers and probes: (1) γ34.5-specific primers: forward 5′-*GGGCTGACCCCTCCCA*-3′, reverse 5′-*TGCTCCGCGGTGACG*-3′, probe 5′-6-carboxyfluorescein [FAM]-*CCCCTCGCGCCCCT*-3′ (amplicon length, 83 bp). (2) LAT primers and probe: forward 5′-*GGGTGGGCTCGTGTTACAG*-3′; reverse 5′-*GGACGGGTAAGTAACAGAGTCTCTA*-3′; probe 5′-FAM-*ACACCAGCCCGTTCTTT*-3′ (amplicon length = 81 bp). (3) dLAT2.6A-specific primers: TaqMan Assay ID: APWC9MM. (4) gB-specific primers: forward 5′-*AACGCGACGCACATCAAG*-3′, reverse 5′-*CTGGTACGCGATCAGAAAGC*-3′, probe 5′-6-carboxyfluorescein [FAM]-*CAGCCGCAGTACTACC*-3′; (5) ICP0-specific primers: forward 5′-*CGGACACGGAACTGTTCGA*-3′; reverse 5′-*CGCCCCCGCAACTG*-3′; probe 5′-FAM-*CCCCATCCACGCCCTG*-3′. and (6) ICP4-specific primers forward, 5′-*GCGTCGTCGAGGTCGT*-3′, reverse 5′-*CGCGGAGACGGAGGAG*-3′; probe, 5′-FAM-*CACGACCCCGACCACC*-3′ (amplicon length, 69 bp). Glyceraldehyde-3-phosphate dehydrogenase (GAPDH) primers (ASSAY I.D. m999999.15_G1; amplicon length, 107 bp; Applied Biosystems) were used as an internal control in all experiments.

### 5.7. Isolation of RNA from TG, Brainstem, Brain, and the Whole Eye of Infected Mice

IFNαβR^−/−^ mice were infected ocularly in both eyes with 1 × 10^2^ pfu/eye of HSV-1 strain LAT-plus or LAT-minus viruses as described above. On days 3 and 5 PI, mice were euthanized and the TG, brainstems, brains, and whole eyes were isolated and homogenized individually as we have described previously [97,99,100]. Following RNA extraction, 700 ng of total RNA was reverse-transcribed as we described above. Primers and probes used to determine expression levels of γ34.5 and gB are described above.

### 5.8. Statistical Analysis

Student’s t tests, one-way analysis of variance (ANOVA), and Tukey’s multiple comparison tests were performed using the computer program Instat (GraphPad, San Diego, CA, USA). Results were considered statistically significant when the “*p*” value was <0.05.

## Figures and Tables

**Table 1:** Survival of mice infected with different doses of McKrae (LAT-plus) and dLAT2903 (LAT-minus) viruses.

	Survival/Total			
	PFU/Eye			
	IFN-αβR^−/−^			WT
Virus strain	1 × 10^4^	1 × 10^3 b^	1 × 10^2 b^	1 × 10^4 b^
McKrae (LAT-plus)	0/5 (0%)	9/16 (56%) ^b^	10/10 (100%) ^b^	10/10 (100%)
dLAT2903 (LAT-minus)	0/5 (0%)	1/16 (6%) ^b^	0/10 (0%) ^b^	10/10 (100%)

^a^ IFNαβR^−/−^ and WT mice were infected ocularly with the specified dose of each virus, and survival was monitored for 28 days. ^b^ Experiments were repeated twice.

**Table 2:** Survival of mice infected with recombinant viruses containing truncated regions of 2 kb LAT.

	Survival/Total			
	PFU/Eye			
	IFN-αβR^−/−^			WT
Virus strain	1 × 10^4^	1 × 10^3^	1 × 10^2^	1 × 10^4^
dLAT3.3A (LAT1.5kb)	0/5 (0%)	3/9 (33%) ^b^	9/9 (100%) ^b^	5/5 (100%)
dLAT2.6A (LAT811bp)	0/5 (0%)	0/8 (0%) ^b^	0/10 (0%) ^b^	5/5 (100%)
dLAT2903-γ34.5null (LAT-γ34.5 minus)	0/5 (0%)	2/5 (40%)	5/5 (100%)	5/5 (100%)

^a^ IFNαβR^−/−^ and WT mice were infected ocularly with the specified dose of each virus and survival was monitored for 28 days. ^b^ Experiments were repeated twice.

## Data Availability

The original contributions presented in this study are included in the article. Further inquiries can be directed to the corresponding author(s).

## References

[1] Dawson C.R. (1984). Ocular herpes simplex virus infections. Clin. Dermatol..

[2] Wilhelmus K.R., Dawson C.R., Barron B.A., Bacchetti P., Gee L., Jones D.B., Kaufman H.E., Sugar J., Hyndiuk R.A., Laibson P.R. (1996). Risk factors for herpes simplex virus epithelial keratitis recurring during treatment of stromal keratitis or iridocyclitis. Herpetic Eye Disease Study Group. Br. J. Ophthalmol..

[3] Stevens J.G. (1989). Human herpesviruses: A consideration of the latent state. Microbiol. Rev..

[4] Rock D.L., Nesburn A.B., Ghiasi H., Ong J., Lewis T.L., Lokensgard J.R., Wechsler S.L. (1987). Detection of latency-related viral RNAs in trigeminal ganglia of rabbits latently infected with herpes simplex virus type 1. J. Virol..

[5] Fraser N.W., Valyi-Nagy T. (1993). Viral, neuronal and immune factors which may influence herpes simplex virus (HSV) latency and reactivation. Microb. Pathog..

[6] Hill J.M., Sedarati F., Javier R.T., Wagner E.K., Stevens J.G. (1990). Herpes simplex virus latent phase transcription facilitates in vivo reactivation. Virology.

[7] Wechsler S.L., Nesburn A.B., Watson R., Slanina S., Ghiasi H. (1988). Fine mapping of the major latency-related RNA of herpes simplex virus type 1 in humans. J. Gen. Virol..

[8] Wechsler S.L., Nesburn A.B., Watson R., Slanina S.M., Ghiasi H. (1988). Fine mapping of the latency-related gene of herpes simplex virus type 1: Alternative splicing produces distinct latency-related RNAs containing open reading frames. J. Virol..

[9] Stevens J.G., Wagner E.K., Devi-Rao G.B., Cook M.L., Feldman L.T. (1987). RNA complementary to a herpesvirus alpha gene mRNA is prominent in latently infected neurons. Science.

[10] Deatly A.M., Spivack J.G., Lavi E.H., O’Boyle 2nd D.R., Fraser N.W. (1988). Latent herpes simplex virus type 1 transcripts in peripheral and central nervous system tissues of mice map to similar regions of the viral genome. J. Virol..

[11] Perng G.C., Dunkel E.C., Geary P.A., Slanina S.M., Ghiasi H., Kaiwar R., Nesburn A.B., Wechsler S.L. (1994). The latency-associated transcript gene of herpes simplex virus type 1 (HSV-1) is required for efficient in vivo spontaneous reactivation of HSV-1 from latency. J. Virol..

[12] Perng G.C., Ghiasi H., Slanina S.M., Nesburn A.B., Wechsler S.L. (1996). The spontaneous reactivation function of the herpes simplex virus type 1 LAT gene resides completely within the first 1.5 kilobases of the 8.3- kilobase primary transcript. J. Virol..

[13] Perng G.C., Slanina S.M., Ghiasi H., Nesburn A.B., Wechsler S.L. (1996). A 371-nucleotide region between the herpes simplex virus type 1 (HSV-1) LAT promoter and the 2-kilobase LAT is not essential for efficient spontaneous reactivation of latent HSV-1. J. Virol..

[14] Perng G.C., Jones C., Ciacci-Zanella J., Stone M., Henderson G., Yukht A., Slanina S.M., Hofman F.M., Ghiasi H., Nesburn A.B. (2000). Virus-induced neuronal apoptosis blocked by the herpes simplex virus latency-associated transcript. Science.

[15] Henderson G., Peng W., Jin L., Perng G.C., Nesburn A.B., Wechsler S.L., Jones C. (2002). Regulation of caspase 8- and caspase 9-induced apoptosis by the herpes simplex virus type 1 latency-associated transcript. J. Neurovirol..

[16] Perng G.C., Maguen B., Jin L., Mott K.R., Osorio N., Slanina S.M., Yukht A., Ghiasi H., Nesburn A.B., Inman M. (2002). A gene capable of blocking apoptosis can substitute for the herpes simplex virus type 1 latency-associated transcript gene and restore wild-type reactivation levels. J. Virol..

[17] Jin L., Perng G.C., Mott K.R., Osorio N., Naito J., Brick D.J., Carpenter D., Jones C., Wechsler S.L. (2005). A herpes simplex virus type 1 mutant expressing a baculovirus inhibitor of apoptosis gene in place of latency-associated transcript has a wild-type reactivation phenotype in the mouse. J. Virol..

[18] Peng W., Henderson G., Inman M., BenMohamed L., Perng G.C., Wechsler S.L., Jones C. (2005). The locus encompassing the latency-associated transcript of herpes simplex virus type 1 interferes with and delays interferon expression in productively infected neuroblastoma cells and trigeminal Ganglia of acutely infected mice. J. Virol..

[19] Jin L., Perng G.C., Carpenter D., Mott K.R., Osorio N., Naito J., Brick D.J., Jones C., Wechsler S.L. (2007). Reactivation phenotype in rabbits of a herpes simplex virus type 1 mutant containing an unrelated antiapoptosis gene in place of latency-associated transcript. J. Neurovirol..

[20] Branco F.J., Fraser N.W. (2005). Herpes simplex virus type 1 latency-associated transcript expression protects trigeminal ganglion neurons from apoptosis. J. Virol..

[21] Perng G.C., Jones C. (2010). Towards an understanding of the herpes simplex virus type 1 latency-reactivation cycle. Interdiscip. Perspect. Infect. Dis..

[22] Allen S.J., Rhode-Kurnow A., Mott K.R., Jiang X., Carpenter D., Rodriguez-Barbosa J.I., Jones C., Wechsler S.L., Ware C.F., Ghiasi H. (2014). Interactions between herpesvirus entry mediator (tnfrsf14) and latency-associated transcript during herpes simplex virus 1 latency. J. Virol..

[23] Tormanen K., Allen S., Mott K.R., Ghiasi H. (2019). The Latency-Associated Transcript Inhibits Apoptosis via Downregulation of Components of the Type I Interferon Pathway during Latent Herpes Simplex Virus 1 Ocular Infection. J. Virol..

[24] Garcia-Sastre A., Biron C.A. (2006). Type 1 interferons and the virus-host relationship: A lesson in detente. Science.

[25] Theofilopoulos A.N., Baccala R., Beutler B., Kono D.H. (2005). Type I interferons (alpha/beta) in immunity and autoimmunity. Annu. Rev. Immunol..

[26] McNab F., Mayer-Barber K., Sher A., Wack A., O’Garra A. (2015). Type I interferons in infectious disease. Nat. Rev. Immunol..

[27] Bowie A.G., Unterholzner L. (2008). Viral evasion and subversion of pattern-recognition receptor signalling. Nat. Rev. Immunol..

[28] Navratil V., de Chassey B., Meyniel L., Pradezynski F., Andre P., Rabourdin-Combe C., Lotteau V. (2010). System-level comparison of protein-protein interactions between viruses and the human type I interferon system network. J. Proteome. Res..

[29] Lin R.J., Liao C.L., Lin E., Lin Y.L. (2004). Blocking of the alpha interferon-induced Jak-Stat signaling pathway by Japanese encephalitis virus infection. J. Virol..

[30] Pestka S., Krause C.D., Walter M.R. (2004). Interferons, interferon-like cytokines, and their receptors. Immunol. Rev..

[31] Pestka S. (2000). The human interferon alpha species and receptors. Biopolymers.

[32] Hardy M.P., Owczarek C.M., Trajanovska S., Liu X., Kola I., Hertzog P.J. (2001). The soluble murine type I interferon receptor Ifnar-2 is present in serum, is independently regulated, and has both agonistic and antagonistic properties. Blood.

[33] Hardy M.P., Sanij E.P., Hertzog P.J., Owczarek C.M. (2003). Characterization and transcriptional analysis of the mouse Chromosome 16 cytokine receptor gene cluster. Mamm. Genome.

[34] de Weerd N.A., Samarajiwa S.A., Hertzog P.J. (2007). Type I interferon receptors: Biochemistry and biological functions. J. Biol. Chem..

[35] Brierley M.M., Fish E.N. (2002). Review: IFN-alpha/beta receptor interactions to biologic outcomes: Understanding the circuitry. J. Interferon. Cytokine Res..

[36] Thomas C., Moraga I., Levin D., Krutzik P.O., Podoplelova Y., Trejo A., Lee C., Yarden G., Vleck S.E., Glenn J.S. (2011). Structural linkage between ligand discrimination and receptor activation by type I interferons. Cell.

[37] Mittnacht S., Straub P., Kirchner H., Jacobsen H. (1988). Interferon treatment inhibits onset of herpes simplex virus immediate- early transcription. Virology.

[38] Mikloska Z., Cunningham A.L. (2001). Alpha and gamma interferons inhibit herpes simplex virus type 1 infection and spread in epidermal cells after axonal transmission. J. Virol..

[39] Leib D.A., Harrison T.E., Laslo K.M., Machalek M.A., Moorman N.J., Virgin H.W. (1999). Interferons regulate the phenotype of wild-type and mutant herpes simplex viruses in vivo. J. Exp. Med..

[40] De Regge N., Van Opdenbosch N., Nauwynck H.J., Efstathiou S., Favoreel H.W. (2010). Interferon alpha induces establishment of alphaherpesvirus latency in sensory neurons in vitro. PLoS ONE.

[41] Mossman K.L., Macgregor P.F., Rozmus J.J., Goryachev A.B., Edwards A.M., Smiley J.R. (2001). Herpes simplex virus triggers and then disarms a host antiviral response. J. Virol..

[42] Leib D.A. (2002). Counteraction of interferon-induced antiviral responses by herpes simplex viruses. Curr. Top Microbiol. Immunol..

[43] Hwang S.Y., Hertzog P.J., Holland K.A., Sumarsono S.H., Tymms M.J., Hamilton J.A., Whitty G., Bertoncello I., Kola I. (1995). A null mutation in the gene encoding a type I interferon receptor component eliminates antiproliferative and antiviral responses to interferons alpha and beta and alters macrophage responses. Proc. Natl. Acad. Sci. USA.

[44] Muller U., Steinhoff U., Reis L.F., Hemmi S., Pavlovic J., Zinkernagel R.M., Aguet M. (1994). Functional role of type I and type II interferons in antiviral defense. Science.

[45] Platanias L.C., Uddin S., Colamonici O.R. (1994). Tyrosine phosphorylation of the alpha and beta subunits of the type I interferon receptor. Interferon-beta selectively induces tyrosine phosphorylation of an alpha subunit-associated protein. J. Biol. Chem..

[46] Conrady C.D., Halford W.P., Carr D.J. (2011). Loss of the type I interferon pathway increases vulnerability of mice to genital herpes simplex virus 2 infection. J. Virol..

[47] Conrady C.D., Thapa M., Wuest T., Carr D.J. (2009). Loss of mandibular lymph node integrity is associated with an increase in sensitivity to HSV-1 infection in CD118-deficient mice. J. Immunol..

[48] Luker G.D., Prior J.L., Song J., Pica C.M., Leib D.A. (2003). Bioluminescence imaging reveals systemic dissemination of herpes simplex virus type 1 in the absence of interferon receptors. J. Virol..

[49] Wang S., Jaggi U., Katsumata M., Ghiasi H. (2024). The importance of IFNalpha2A (Roferon-A) in HSV-1 latency and T cell exhaustion in ocularly infected mice. PLoS Pathog..

[50] Wang S., Jaggi U., Oh J.J., Ghiasi H. (2001). IFNβ absence compensates for LAT functions in latency-reactivation and T cell exhaustion. J. Virol..

[51] Bourdon M., Manet C., Montagutelli X. (2025). 2020. Host genetic susceptibility to viral infections: The role of type I interferon induction. Genes Immun..

[52] Duncan C.J.A., Randall R.E., Hambleton S. (2021). Genetic Lesions of Type I Interferon Signalling in Human Antiviral Immunity. Trends Genet..

[53] Bucciol G., Effort C.H.G., Meyts I. (2023). Inherited and acquired errors of type I interferon immunity govern susceptibility to COVID-19 and multisystem inflammatory syndrome in children. J. Allergy Clin. Immunol..

[54] Sancho-Shimizu V., Perez de Diego R., Jouanguy E., Zhang S.Y., Casanova J.L. (2011). Inborn errors of anti-viral interferon immunity in humans. Curr. Opin. Virol..

[55] Chen S.H., Kramer M.F., Schaffer P.A., Coen D.M. (1997). A viral function represses accumulation of transcripts from productive-cycle genes in mouse ganglia latently infected with herpes simplex virus. J. Virol..

[56] Garber D.A., Schaffer P.A., Knipe D.M. (1997). A LAT-associated function reduces productive-cycle gene expression during acute infection of murine sensory neurons with herpes simplex virus type 1. J. Virol..

[57] Ahmed M., Lock M., Miller C.G., Fraser N.W. (2002). Regions of the herpes simplex virus type 1 latency-associated transcript that protect cells from apoptosis in vitro and protect neuronal cells in vivo. J. Virol..

[58] Yordy B., Iijima N., Huttner A., Leib D., Iwasaki A. (2012). A neuron-specific role for autophagy in antiviral defense against herpes simplex virus. Cell Host Microbe.

[59] Johnson K.E., Song B., Knipe D.M. (2008). Role for herpes simplex virus 1 ICP27 in the inhibition of type I interferon signaling. Virology.

[60] Mossman K.L., Saffran H.A., Smiley J.R. (2000). Herpes simplex virus ICP0 mutants are hypersensitive to interferon. J. Virol..

[61] Kew C., Lui P.Y., Chan C.P., Liu X., Au S.W., Mohr I., Jin D.Y., Kok K.H. (2013). Suppression of PACT-induced type I interferon production by herpes simplex virus 1 Us11 protein. J. Virol..

[62] Cotter C.R., Nguyen M.L., Yount J.S., Lopez C.B., Blaho J.A., Moran T.M. (2010). The virion host shut-off (vhs) protein blocks a TLR-independent pathway of herpes simplex virus type 1 recognition in human and mouse dendritic cells. PLoS ONE.

[63] Wang S., Wang K., Lin R., Zheng C. (2013). Herpes simplex virus 1 serine/threonine kinase US3 hyperphosphorylates IRF3 and inhibits beta interferon production. J. Virol..

[64] Paladino P., Mossman K.L. (2009). Mechanisms employed by herpes simplex virus 1 to inhibit the interferon response. J. Interferon. Cytokine Res..

[65] Chou J., Kern E.R., Whitley R.J., Roizman B. (1990). Mapping of herpes simplex virus-1 neurovirulence to gamma 134.5, a gene nonessential for growth in culture. Science.

[66] McGeoch D.J., Dalrymple M.A., Davison A.J., Dolan A., Frame M.C., McNab D., Perry L.J., Scott J.E., Taylor P. (1988). The complete DNA sequence of the long unique region in the genome of herpes simplex virus type 1. J. Gen. Virol..

[67] Chou J., Roizman B. (1992). The gamma 1(34.5) gene of herpes simplex virus 1 precludes neuroblastoma cells from triggering total shutoff of protein synthesis characteristic of programed cell death in neuronal cells. Proc. Natl. Acad. Sci. USA.

[68] Chou J., Roizman B. (1994). Herpes simplex virus 1 gamma(1)34.5 gene function, which blocks the host response to infection, maps in the homologous domain of the genes expressed during growth arrest and DNA damage. Proc. Natl. Acad. Sci. USA.

[69] Wilcox D.R., Longnecker R. (2016). The Herpes Simplex Virus Neurovirulence Factor gamma34.5: Revealing Virus-Host Interactions. PLoS Pathog..

[70] He B., Gross M., Roizman B. (1997). The gamma(1)34.5 protein of herpes simplex virus 1 complexes with protein phosphatase 1alpha to dephosphorylate the alpha subunit of the eukaryotic translation initiation factor 2 and preclude the shutoff of protein synthesis by double-stranded RNA-activated protein kinase. Proc. Natl. Acad. Sci. USA.

[71] Orvedahl A., Alexander D., Talloczy Z., Sun Q., Wei Y., Zhang W., Burns D., Leib D.A., Levine B. (2007). HSV-1 ICP34.5 confers neurovirulence by targeting the Beclin 1 autophagy protein. Cell Host Microbe.

[72] Verpooten D., Ma Y., Hou S., Yan Z., He B. (2009). Control of TANK-binding kinase 1-mediated signaling by the gamma(1)34.5 protein of herpes simplex virus 1. J. Biol. Chem..

[73] Drolet B.S., Perng G.C., Villosis R.J., Slanina S.M., Nesburn A.B., Wechsler S.L. (1999). Expression of the first 811 nucleotides of the herpes simplex virus type 1 latency-associated transcript (LAT) partially restores wild-type spontaneous reactivation to a LAT-null mutant. Virology.

[74] Samoto K., Perng G.C., Ehtesham M., Liu Y., Wechsler S.L., Nesburn A.B., Black K.L., Yu J.S. (2001). A herpes simplex virus type 1 mutant deleted for gamma34.5 and LAT kills glioma cells in vitro and is inhibited for in vivo reactivation. Cancer Gene Ther..

[75] Ghiasi H., Kaiwar R., Nesburn A.B., Wechsler S.L. (1992). Expression of herpes simplex virus type 1 glycoprotein B in insect cells. Initial analysis of its biochemical and immunological properties. Virus Res..

[76] Mador N., Goldenberg D., Cohen O., Panet A., Steiner I. (1998). Herpes simplex virus type 1 latency-associated transcripts suppress viral replication and reduce immediate-early gene mRNA levels in a neuronal cell line. J. Virol..

[77] Peng W., Vitvitskaia O., Carpenter D., Wechsler S.L., Jones C. (2008). Identification of two small RNAs within the first 1.5-kb of the herpes simplex virus type 1-encoded latency-associated transcript. J. Neurovirol..

[78] Tormanen K., Matundan H.H., Wang S., Jaggi U., Mott K.R., Ghiasi H. (2022). Small Noncoding RNA (sncRNA1) within the Latency-Associated Transcript Modulates Herpes Simplex Virus 1 Virulence and the Host Immune Response during Acute but Not Latent Infection. J. Virol..

[79] Tormanen K., Wang S., Matundan H.H., Yu J., Jaggi U., Ghiasi H. (2022). Herpes Simplex Virus 1 Small Noncoding RNAs 1 and 2 Activate the Herpesvirus Entry Mediator Promoter. J. Virol..

[80] Oh J.J., Jaggi U., Tormanen K., Wang S., Hirose S., Ghiasi H. (2024). The anti-apoptotic function of HSV-1 LAT in neuronal cell cultures but not its function during reactivation correlates with expression of two small non-coding RNAs, sncRNA1&2. PLoS Pathog..

[81] Jurak I., Kramer M.F., Mellor J.C., van Lint A.L., Roth F.P., Knipe D.M., Coen D.M. (2010). Numerous conserved and divergent microRNAs expressed by herpes simplex viruses 1 and 2. J. Virol..

[82] Umbach J.L., Kramer M.F., Jurak I., Karnowski H.W., Coen D.M., Cullen B.R. (2008). MicroRNAs expressed by herpes simplex virus 1 during latent infection regulate viral mRNAs. Nature.

[83] Cui C., Griffiths A., Li G., Silva L.M., Kramer M.F., Gaasterland T., Wang X.J., Coen D.M. (2006). Prediction and identification of herpes simplex virus 1-encoded microRNAs. J. Virol..

[84] Kramer M.F., Jurak I., Pesola J.M., Boissel S., Knipe D.M., Coen D.M. (2011). Herpes simplex virus 1 microRNAs expressed abundantly during latent infection are not essential for latency in mouse trigeminal ganglia. Virology.

[85] Tang S., Bertke A.S., Patel A., Wang K., Cohen J.I., Krause P.R. (2008). An acutely and latently expressed herpes simplex virus 2 viral microRNA inhibits expression of ICP34.5, a viral neurovirulence factor. Proc. Natl. Acad. Sci. USA.

[86] Farrell M.J., Dobson A.T., Feldman L.T. (1991). Herpes simplex virus latency-associated transcript is a stable intron. Proc. Natl. Acad. Sci. USA.

[87] Cliffe A.R., Garber D.A., Knipe D.M. (2009). Transcription of the herpes simplex virus latency-associated transcript promotes the formation of facultative heterochromatin on lytic promoters. J. Virol..

[88] Phelan D., Barrozo E.R., Bloom D.C. (2017). HSV1 latent transcription and non-coding RNA: A critical retrospective. J. Neuroimmunol..

[89] Ghiasi H., Cai S., Perng G.C., Nesburn A.B., Wechsler S.L. (2000). Both CD4+ and CD8+ T cells are involved in protection against HSV-1 induced corneal scarring. Br. J. Ophthalmol..

[90] Erlich K.S., Wofsy D., Dix R.D., Mills J. (1989). Effects of selective depletion of L3T4+ T-lymphocytes on herpes simplex virus encephalitis. Clin. Immunol. Immunopathol..

[91] Newell C.K., Martin S., Sendele D., Mercadal C.M., Rouse B.T. (1989). Herpes simplex virus-induced stromal keratitis: Role of T-lymphocyte subsets in immunopathology. J. Virol..

[92] Newell C.K., Sendele D., Rouse B.T. (1989). Effects of CD4+ and CD8+ T-lymphocyte depletion on the induction and expression of herpes simplex stromal keratitis. Reg. Immunol..

[93] Wang S., Ljubimov A.V., Jin L., Pfeffer K., Kronenberg M., Ghiasi H. (2018). Herpes Simplex Virus 1 Latency and the Kinetics of Reactivation Are Regulated by a Complex Network of Interactions between the Herpesvirus Entry Mediator, Its Ligands (gD, BTLA, LIGHT, and CD160), and the Latency-Associated Transcript. J. Virol..

[94] Mott K., Brick D.J., van Rooijen N., Ghiasi H. (2007). Macrophages Are Important Determinants of Acute Ocular HSV-1 Infection in Immunized Mice. Investig. Ophthalmol. Vis. Sci..

[95] Allen S.J., Hamrah P., Gate D.M., Mott K.R., Mantopoulos D., Zheng L., Town T., Jones C., von Andrian U.H., Freeman G.J. (2011). The role of LAT in increased CD8+ T cell exhaustion in trigeminal ganglia of mice latently infected with herpes simplex virus type 1. J. Virol..

[96] Osorio Y., Ghiasi H. (2003). Comparison of adjuvant efficacy of herpes simplex virus type 1 recombinant viruses expressing TH1 and TH2 cytokine genes. J. Virol..

[97] Mott K.R., Perng G.C., Osorio Y., Kousoulas K.G., Ghiasi H. (2007). A Recombinant Herpes Simplex Virus Type 1 Expressing Two Additional Copies of gK Is More Pathogenic than Wild-Type Virus in Two Different Strains of Mice. J. Virol..

[98] Jaggi U., Matundan H.H., Tormanen K., Wang S., Yu J., Mott K.R., Ghiasi H. (2020). Expression of Murine CD80 by Herpes Simplex Virus 1 in Place of Latency-Associated Transcript (LAT) Can Compensate for Latency Reactivation and Anti-apoptotic Functions of LAT. J. Virol..

[99] Ghiasi H., Osorio Y., Perng G.C., Nesburn A.B., Wechsler S.L. (2002). Overexpression of interleukin-2 by a recombinant herpes simplex virus type 1 attenuates pathogenicity and enhances antiviral immunity. J. Virol..

[100] Mott K.R., Osorio Y., Brown D.J., Morishige N., Wahlert A., Jester J.V., Ghiasi H. (2007). The corneas of naive mice contain both CD4+ and CD8+ T cells. Mol. Vis..

